# Visuo-Spatial Working Memory and Mathematical Skills in Children: A Network Analysis Study

**DOI:** 10.3390/bs13040294

**Published:** 2023-03-30

**Authors:** Luigi Macchitella, Giorgia Tosi, Daniele Luigi Romano, Marika Iaia, Francesca Vizzi, Irene C. Mammarella, Paola Angelelli

**Affiliations:** 1 Scientific Institute I.R.C.C.S. “E. Medea”, Unit for Severe Disabilities in Developmental Age and Young Adults (Developmental Neurology and Neurorehabilitation), 72100 Brindisi, Italy; 2 Department of Psychology, University of Milano-Bicocca, 20126 Milan, Italy; giorgia.tosi@unimib.it (G.T.); daniele.romano@unimib.it (D.L.R.); 3 Lab of Applied Psychology and Intervention, Department of Human and Social Studies, University of Salento, 73100 Lecce, Italy; marika.iaia@unisalento.it (M.I.); francesca.vizzi@unisalento.it (F.V.); paola.angelelli@unisalento.it (P.A.); 4 Department of Developmental and Social Psychology, University of Padova, 35100 Padova, Italy; irene.mammarella@unipd.it

**Keywords:** visuo-spatial working memory, mathematical abilities, Network Analysis

## Abstract

Visuo-spatial working memory is one of the main domain-general cognitive mechanisms underlying mathematical abilities and their development in children. However, if visuo-spatial working memory involves different processes and components, then the term ‘mathematics’ refers to a broad concept that includes multiple domains and skills. The aim of this present study was to investigate the relationship between different visuo-spatial working memory components and several mathematical abilities in a sample of third- to fifth-grade Italian children. To assess the relationships between different visuo-spatial working memory components and different mathematical abilities, we relied on Network Analysis (NA). Results indicate that some but not all visuo-spatial working memory components are associated with some mathematical abilities.

## 1. Introduction

Studying the neurocognitive basis of mathematical thinking is important for several reasons. First, it allows us to acquire information regarding the evolution, development, and functional organization of neurocognitive abilities that are unique in humans and reach the maximum expression in our species (e.g., complex arithmetic skills, as well as reading skills) [1]. Moreover, mathematical cognition supports essential skills in daily life and allows us to make sense of the world around us [2,3].

In the literature, there is a debate about whether numerical cognition involves specialized neurocognitive mechanisms or arises from domain-general cognitive processes [4,5,6,7,8]. However, both neuroscientific and cognitive research points out that cognitive mechanisms underlying mathematical abilities overlap (i.e., share some brain and cognitive basis) [6,9,10] and, in any case, interact [11,12] with other domain-general neurocognitive processes, such as memory, attention, working memory, language, and motor and spatial functions. Several studies have shown that visuo-spatial working memory (VSWM) is one of the main domain-general cognitive mechanisms underlying mathematical abilities and their development in children [13,14,15]. Notably, if the VSWM involves different processes and components (see below), then it is also important to take into account that the term ‘mathematics’ refers to a broad concept that includes multiple domains (e.g., basic number knowledge, geometry, and arithmetic). These domains can further be divided into several subdomains (e.g., written calculation), as well as into more specific skills (e.g., retrieval of arithmetical facts, maintaining intermediate results, carrying procedures, or specific operations such as addition, subtraction, multiplication and division). Thus, it is possible that, in children, diverse mathematical abilities have different relationships with the various VSWM components [16,17].

The aim of this present study was to investigate the relationship between the different VSWM components and several mathematical abilities in a sample of third- to fifth-grade Italian children who are at a developmental stage in which the rudiments of mathematics have largely been acquired. As we were interested in having a large spread of performance in all of the critical measures, we recruited a group of children who attended school regularly and excluded only children with very low non-verbal intelligence. To test the relationships between VSWM components and the mathematical abilities model, we relied on Network Analysis (NA). This is a flexible method for exploring the relationships among various domains when the main interest is the direct relationship among a set of variables that are intercorrelated. A brief presentation of working memory models and studies concerning VSWM and mathematical abilities are reported below.

### 1.1. Working Memory Model(s)

Working memory (WM) refers to a system with limited capacity that allows temporary storage, processing, and manipulation of information while performing complex cognitive activities [18]. One of the most influential models of WM was formulated by Baddeley [19]. In its current version, the WM model comprises four main subcomponents (for a review, see [18,19,20,21,22]). The phonological loop and the visuo-spatial sketchpad are two modality-specific subsystems (called short-term memory systems; STM) that are responsible, respectively, for the passive short-term storage and maintenance of phonological/verbal and visuo-spatial information. STM is assessed via the so-called ‘simple, passive, or low-control STM span tasks’, which require the storage of items in the same format as that in which they are presented. The term WM refers specifically to the Central Executive (CE) component: a domain-general (i.e., modality non-specific) limited capacity system responsible for the active control, manipulation, and governance of processing information within and across the two subsidiary systems. WM is assessed via the so-called ‘complex, active, or high-control WM span tasks’, which require storage of stimuli as well as active manipulation and transformation of information (see, e.g., [23,24,25,26,27]). Finally, the episodic buffer is a limited-capacity system that, for a short time, stores a unitary and multidimensional representation of an event. Baddeley’s WM model postulates a clear distinction between CE (i.e., WM) and the passive storage of information in two subsidiary systems not requiring executive control (i.e., STM) [21] (see also, e.g., [23,25,27,28]). The differentiation between STM and WM acquires importance with regard to this present study, given that some studies indicate that only complex WM span tasks and related cognitive and brain mechanisms (i.e., CE) are predictive of complex cognitive abilities (such as reading comprehension, fluid intelligence and mathematical ability) (see, e.g., [29,30,31], and see below for some data concerning mathematical abilities). The distinction between WM and STM has, however, been reformulated by Cornoldi and Vecchi in terms of a continuum along active and passive processes [27,32]. In particular, their WM model (called the ‘Continuity Model’ of WM) (C-WM) does not postulate a clear distinction between active/high-control WM processes (i.e., CE) and passive/low-control processes that involve STM. Indeed, several studies point out that there is no STM storage without some degree of central executive control (i.e., even passive and ‘simple’ storage recruits some degree of attentional control) [25,32,33,34]. In accordance with C-WM, in this study, we use the term VSWM to refer to the short-term storage, maintenance, and manipulation of visuo-spatial information, which may vary along a continuum in relation to the degree of executive control involved. Thus, we use the terms passive/low-control VSWM and active/high-control VSWM to refer to processes and tasks that involve, respectively, fewer or more executive resources. C-WM stressed two important issues regarding this present study. The first concerns whether CE is a domain-general cognitive control system or whether there are separate central executive resources for different types of information (i.e., a visuo-spatial CE and a verbal CE) [23,26,32,35,36]. The second concerns the distinction of several components within VSWM. According to the C-WM, VSWM is composed of different components, such as a visual (involved in the storage of the shape and/or texture of the stimulus), spatial-sequential (which refers to the storage of spatial locations presented sequentially), and spatial-simultaneous components (devoted to the storage of spatial locations presented simultaneously) [32,37,38,39,40,41,42]. Regarding this present study, the distinction among different VSWM components is important, given that previous studies rarely assessed the relationships between mathematical skills and different VSWM components. 

### 1.2. VSWM and Mathematical Abilities

A huge number of studies carried out on adults, typically developing (TD) children, and children with a mathematical learning disability (MLD) have clearly shown that VSWM, and, more generally, WM, plays a critical role in mathematical skills and their development for a meta-analysis (for a meta-analysis see, e.g., [13,43,44]). Although an association between VSWM and mathematical skills has been clearly demonstrated, there is still a lack of consensus on several questions. A first open question concerns the importance of the different WM processes (i.e., passive/low-control processes and active/high-control processes) on mathematical skills. Several sets of data indicate that CE (i.e., active/high-control processes) plays a key role in mathematical skills (from early numerical abilities to arithmetic skills), as well as in mathematical difficulty [44,45,46,47,48,49]. This evidence is consistent with the data showing that only CE seems to be associated with individual differences in complex cognitive skills such as mathematical abilities (see above references). In contrast, other studies suggest that low-control VSWM processes, but not high-control WM processes, are connected with mathematical skills [50,51]. Finally, there are also studies suggesting that both low- and high-control VSWM processes may be associated with mathematical abilities and difficulties [52,53,54,55,56,57,58,59,60].

Moreover, there is still a debate over whether all or only some of the passive VSWM components (the visual, spatial-sequential, and spatial-simultaneous components) are associated with mathematical skills (see discussion). In addition to the open questions concerning the relative importance of the different VSWM processes and components in mathematical abilities, other data draw a very complex picture of associations between VSWM and mathematics. Indeed, previous evidence shows that the contribution of the different WM components and processes to mathematical skills may change depending on the mathematical domains considered, as well as on the children’s age or expertise regarding specific mathematical tasks [17,53,61,62]. For example, a development-related shift from a visuo-spatial to a verbal WM-related strategy in solving mathematical tasks has been proposed. In more detail, some data suggest that VSWM plays a key role in young children in solving mathematical tasks and, more generally, in supporting the acquisition of new mathematical skills, while in older children (and/or when a mathematical ability has been acquired), mathematical tasks mainly involve verbal strategies related to verbal WM see [17,61,62]. Accordingly, several pieces of research indicate that during primary school, the association between VSWM and mathematical ability declines from around 7–8 years [17,53,55,62]. Notably, however, the data regarding a development-related shift from a visuo-spatial to a verbal strategy are strongly inconsistent in the literature (see the discussion below).

Finally, the contribution of each WM component in a mathematical domain may change depending on several other factors. For example, in the calculation, the contribution of the different WM components may depend on the modality (e.g., visual or auditory) and the format (e.g., horizontal or vertical) through which operations are presented (see, e.g., [2,39,63]). It has been suggested that VSWM is mainly associated with tasks involving the processing of the analogical representation of quantities, such as in number comparison tasks and in several mathematical domains that clearly rely on visual processing skills (e.g., geometry or written calculation) [16,57,64,65,66,67].

### 1.3. The Present Study

The association between VSWM and mathematical skills has repeatedly been shown. However, what is not yet clear is the relative importance and the different contribution of specific VSWM processes (i.e., active/high-control vs. passive/low-control processes) and components (visual, spatial-sequential, and spatial-simultaneous components) to different mathematical abilities [53]. Regarding the different components of VSWM, several authors have pointed to the need for more studies disclosing the relationship between mathematical learning and the specific subcomponents of VSWM in TD children [13,58,68]. Many studies, for instance, do not make a separate assessment of passive visual WM and passive spatial-simultaneous WM [16,56,69] (see also the references in the meta-analysis of [13]). Concerning active VSWM, as also stressed by other authors [68], many studies have investigated the connection between CE and mathematics without taking into account separate central executive resources for different types of information (i.e., verbal vs. visuo-spatial), collapsing performances on verbal and visuo-spatial active WM tasks into a unique score [48,49,51,55,67,70]. However, some data suggest that active VSWM, but not active verbal WM, may be associated with mathematical abilities (and disabilities) [15,60,66,71]. Finally, several studies assess the relationship between VSWM and general mathematical domains and abilities (e.g., ‘numerical operations’ or ‘early mathematical knowledge’) without taking into account the associations between VSWM and more specific mathematical skills (e.g., counting ability, mental calculation, written calculation) [53,68,70].

Overall, the primary aim of this present study is to assess the associations between the different processes (active/high-control and passive/low-control processes) and components (visual, spatial-sequential, and spatial-simultaneous) of VSWM and different mathematical skills, including understanding the semantic and syntactic structure of numbers, written calculation, and arithmetical facts. 

Notably, although this present study was not designed to assess age- and development-related changes in the correlation strength between VSWM and mathematical abilities, we assessed children (ranging in age from 7.9 to 11.2 years) attending the third to the fifth grades of primary school. 

Crucially, in order to assess the associations between mathematical abilities and different VSWM processes and components, it is important to take into account the influence that high-control WM processes may have on passive WM tasks (and vice versa). For instance, to assess the possibility that it is not only active VSWM but also passive VSWM, that is involved in mathematical abilities, it is important to consider that (according to C-WM), passive and active span tasks cannot be clearly differentiated and that there are no passive tasks that have no degree of central executive control. Indeed, C-WM does not postulate a clear distinction between simple and complex tasks: ‘it is clearly impossible to define a task as being completely passive or completely active. Every task has a degree of active control, even if minimal (as in case of very passive storage tasks)’ [32]. Thus, an association between passive VSWM and mathematical skills, or an association between both passive and active VSWM and mathematical skills, could still be interpreted under the hypothesis that the correlations between passive VSWM and mathematical skills arise, to some extent, by the influence of active/high-control VSWM on mathematics and/or on passive VSWM tasks. Moreover, in order to explain the involvement of different VSWM components (visual, spatial-sequential, and spatial-simultaneous) in mathematical abilities, it is also important to consider the possibility that various components may involve different levels of executive control (see [37,72]). In order to address these issues, in this study, we use a statistical method, Network Analysis, which is a flexible method for exploring the relationships among various domains when the main interest is the direct relationships among a set of variables that are intercorrelated. Indeed, Network Analysis is a method with high specificity to reveal the direct correlation between two variables net of the possible influence (i.e., variance explained) of other variables (see below for details). Even if Network Analysis may miss some significant correlations (false negatives), it is a method with high specificity (few false positives).

## 2. Materials and Methods

### 2.1. Participants

A sample of 118 Italian children (50 females and 68 males), ranging in age from 7.9 to 11.2 years (average age: 9.80 ± 0.80), were recruited from 3 primary schools in southern Italy. The only exclusion criterion was performance on Raven’s Colored Progressive Matrices [73] below the normative values (at least SDs) based on Italian norms [74]. In particular, 24 3rd-grade children (average age: 8.55 ± 0.04), 41 4th-grade children (average age: 9.51 ± 0.35), and 53 5th-grade children (average age: 10.58 ± 0.30) participated in this study. All the children attended school regularly, and none were singled out by their teachers as being socio-economically disadvantaged. This study was performed in schools in southern Italy, in areas without major migratory flows and devoted to the primary and secondary economic sectors. The parents were informed about the research activities and authorized their child’s participation by furnishing written informed consent. This study was conducted according to the principles of the Helsinki Declaration and approved by the school authorities and the Ethics Committee of Psychological Research of the Department of Human and Social Studies, University of Salento (Prot. 101206—29 July 2020).

### 2.2. Neuropsychological Tests

#### 2.2.1. Mathematical Tasks

The AC-MT Battery [75] was used to assess the children’s mathematical skills. The children were presented with four mathematical tasks, controlled for difficulty (according to their school grade). The tasks used in this present work are described below:

- Written calculation. This subtest assesses the child’s ability to complete written computational operations (addition, subtraction, multiplication, and division);
- Number ordering. In this task, the child orders number sequences from the lowest to the highest and vice versa. This task requires an understanding of the semantics of numbers to place the numbers in the correct order;
- Transcoding. This task assesses students’ ability to process the syntactic structure of a number. Students are shown 6 series of verbally described numbers (e.g., 3 tens, 8 units, and 2 hundreds) and are asked to transform them into a final number (i.e., 238);
- Arithmetical Facts. This task is used to investigate how children have stored combinations of numbers and whether they are able to access them automatically. The items include simple additions, subtractions, and multiplications and are presented verbally and allowing 5 s to answer for each of the 12 items.

In Arithmetic Facts task, errors were recorded, while in all other tests, correct answers were collected. For analysis purposes, we reversed the Arithmetic Facts score.

#### 2.2.2. VSWM Tasks

We assessed both passive and active VSWM through five subtests included in the Visuospatial Working Memory Test Battery [76]. Four tests were used to assess passive VSWM, while one test was used to assess active VSWM. Active VSWM was assessed via active version of visual pattern task (A-VPT). A-VPT requires memorizing a pattern of positions in a matrix; after that, the children are required to reproduce it on an empty matrix but shift the original pattern one line below. Three computerized recognition tasks were used to assess passive visual WM (i.e., “balloon recognition task”), passive spatial-simultaneous WM (“simultaneous dot task”), and passive spatial-sequential WM (“sequential matrix task”). The three recognition tasks had the same structure: children were asked to decide if a series of sequential locations (“sequential matrix task”) or pattern of location (in “simultaneous dot task”) or figures with a different texture (in the balloon recognition task) were the same or different from the one previously presented. All the tasks used in this study have been used in other studies for a detailed description of the tasks (see, e.g., [37,57,58]). Finally, we also used the Corsi Blocks Test included in Visuospatial Working Memory Test battery to assess passive spatial-sequential WM [76].

### 2.3. Statistical Analyses

The z-scores of the neuropsychological tests were used as variables.

We explored the associations between mathematical skills and working memory domains by adopting a basic correlation approach followed by a Network Analysis approach.

Pearson’s correlations were used for the basic correlation analysis. To highlight direct non-spurious correlations, we adopted a Gaussian Graphical Model (see below for the detail). 

The analyses were performed using JASP [77], which bases the network modules on the bootnet [78] and qgraph [79] packages of the R statistical software [80].

#### Network Analysis

An interesting statistical method for exploring the relationships between variables is Network Analysis (NA). A network is formed by a set of nodes (i.e., the variables of interest) and a set of edges connecting those nodes (i.e., their relationships). In recent years, NA has been used for studying patients’ neuropsychological profiles [81,82,83,84], comorbidity in learning skills [85,86], psychopathology and personality traits [87,88], and body illusions [89,90].

In psychology, an approach that is frequently used to estimate networks is the Gaussian Graphical Model (GGM), in which the edges represent regularized partial correlation coefficients between two variables after conditioning on all other variables in the dataset [78]. GGM is a specification of the Pairwise Markov Random Field (PMRF), a model in which undirected edges indicate conditional dependence between two variables [87,88]. The regularization of correlation coefficients adopted in GGM is the ‘least absolute shrinkage and selection operator’ (LASSO) algorithm [91]. Such penalty shrinks to zero small connections [78]. LASSO regularization utilizes a tuning parameter, which controls for the sparsity of the network (i.e., the presence of 0-value correlations) [92]. The selection of the best tuning parameter is based on the Extended Bayesian Information Criterion (EBIC), which uses a hyperparameter γ that we set at 0.25, as suggested in the literature [92]. As a result, if an edge is not reduced to zero, it implies that it is sufficiently robust to be incorporated in the model, and that edge indicates a conditional dependency between the two nodes.

One way to check for the stability of the resulting network is the bootstrapping procedure, which recurrently estimates the model under resampled data [78]. We resampled 1000 times, obtaining the 95% Confidence Interval (CI) of each edge and the average edge value over the resampling. The CI of each edge provides important information about the replicability of the resulting network. The edges that do not include 0 in their CI are more likely to be replicated; when the CI touches 0, the edge has some chance of not being found in a different data collection, and edges that cross 0 are unreliable because they may have an opposite sign in a different sample [78,86].

We also computed the strength centrality index, which sheds light on the relative importance of each node in the specific estimated network [87]. Strength aims to assess a node’s influence in the network and consists of the sum of the absolute value weights of the edges passing through the node [93].

We included, as the nodes of the network, the z-scores of all the neuropsychological tests used to assess working memory (both passive and active VSWM subtests) and mathematical skills (Arithmetical facts subtest, Written calculation, Number ordering, and Transcoding digits).

## 3. Results

**Figure 1:**
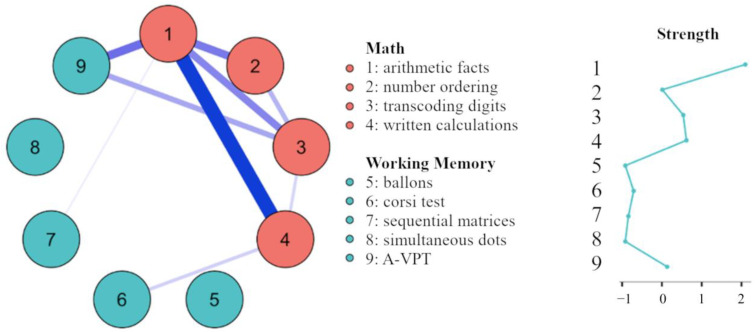
The left panel shows the network estimated by the Gaussian Graphical Model, adopting the EBIC Graphical LASSO method. The edges represent regularized partial correlations. Blue lines indicate positive associations. Red lines would have indicated negative associations (none observed). The size and the color saturation of the edges represent the intensity of the relationships. The nodes indicate the variables as follows: (1) Arithmetical facts; (2) Number ordering; (3) Transcoding digits (4) Written calculation; (5) Balloon recognition task; (6) Corsi test; (7) Sequential matrices; (8) Simultaneous dots; and (9) A-VPT. The right panel shows the centrality index. Strength centrality is higher if a node has a large number of strong direct connections with other nodes. The centrality index is standardized and expressed as z-scores.

The lower triangle of Table 1 reports the simple correlations among all the variables. The results show that most of the working memory tasks are related to specific mathematical skills. In particular, A-VPT is associated with all mathematical tests; the balloons test is associated with both arithmetic facts and written calculation; the Corsi blocks task is associated with number transcoding and written calculation; and sequential matrices are related only to arithmetic facts. Interestingly, after the application of the Network Analysis, fewer direct associations were detected (as shown in the upper triangle of Table 1). In particular, A-VPT is connected with arithmetical facts and number transcoding only, the Corsi blocks task shows a direct relationship with written calculation, and sequential matrices are uniquely related to arithmetical facts. This pattern is shown in Figure 1 (left panel), which represents the best estimated GGM. The network confirms a cluster of mathematical skills, including arithmetical facts, number ordering, transcoding digits, and written calculation. Among these variables, arithmetical facts and written calculations show the strongest connections. This observation is confirmed by the strength centrality index, which suggests that mathematical variables have strong connections inside the network (Figure 1, right panel). On the other hand, the working memory tests seem to catch different cognitive abilities and do not aggregate into a single community, thus showing low strength.

**Figure 2:**
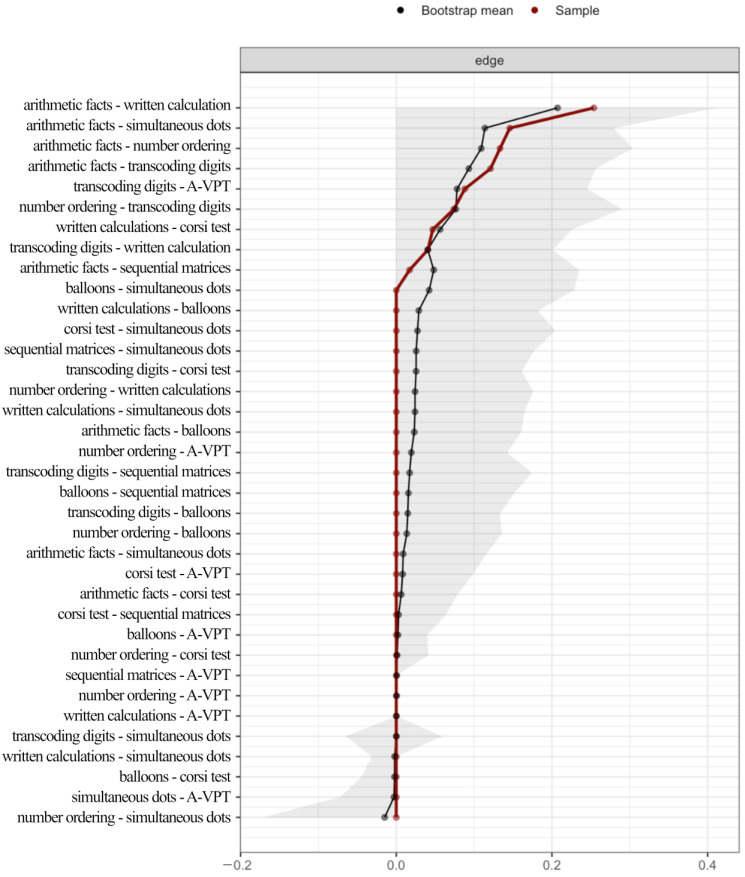
Results from the 1000 bootstraps. Red dots indicate the edge value in the estimated network. Black dots indicate the average edge value over 1000 bootstrap resampling. The grey shadow represents the 95% confidence interval estimated with the bootstrap resampling.

Figure 2 reports the bootstrap results, showing that all the edges that emerged in the Network Analysis are reliable in their direction (positive vs. negative correlations). Among the estimated edges, none crossed 0. As for the strongest edges, the results are somewhat overestimated and do not show a clear overlap between the observed and the bootstrap mean.

## 4. Discussion

Which components and processes of WM correlate to specific math skills is an open question in the literature. The primary aim of this study was to acquire more information concerning the involvement of different VSWM processes (high-control/active and/or low-control/passive) and components (visual, spatial-sequential, and spatial-simultaneous) in different mathematical abilities in an unselected sample of third- to fifth-grade primary schoolchildren. Notably, in our study, we addressed some of the associations which have never been investigated in the literature. Moreover, addressing the associations between VSWM and math through the use of a NA allows us to acquire information that cannot be provided through the analysis of simple correlations and/or other methods. Indeed, NA gives an item-level look into the correlations between variables and highlights the unique association between any two variables after conditioning on all others. The employment of NA on the scores of neuropsychological tests allows the evaluation of how they relate to each other on an item level. On the contrary, simple associations do not allow isolating the specific contribution of single neuropsychological tests net of other associations. Consequently, our results can provide useful, predictive, and interpretable information concerning both treatments of math deficits and neurocognitive models of mathematical skills.

We start by discussing the results regarding the relationship between the different VSWM components/processes and math considered globally, and then we discuss the data on the association between specific mathematical abilities and specific VSWM components/processes.

### 4.1. The Relationship between Mathematical Abilities and Different VSWM Components and Processes

So far, regarding the relative importance of low-and/or high-control VSWM processes for mathematical abilities, several studies have indicated that only CE or active VSWM plays a critical role in mathematics tasks [44,45,46,47,49], while other studies have suggested that passive VSWM is also (or is only) involved in mathematical performance [50,51,52,53,54,55,56,57,58,59]. In this study, we found that both active and passive VSWM play a role in mathematical skills. Indeed, both simple correlations and Network Analysis showed that both active and passive VSWM are associated with mathematical tasks. Notably, thanks to the Network Analysis method, we can suggest that both active and passive VSWM are connected, independently of each other, to mathematical skills.

Concerning the different involvement of the passive VSWM components in mathematical skills, it is not yet clear whether only some components are linked to mathematical abilities and others are not. It has been suggested that, while the visual subcomponent has little (compared with the spatial component) or no influence [58,59], the spatial components (both the spatial-sequential and the spatial-simultaneous components) are involved in mathematical skills [57,58]; however, there are other studies suggesting that only the spatial-simultaneous component plays a critical role ([52], but see [69]).

In this present study, we found that not all passive VSWM components are connected to mathematical abilities. In particular, simple correlations indicate that only visual and spatial-sequential components are associated with mathematical skills, while the passive spatial-simultaneous component is not. However, thanks to the application of the networks analysis we found that only passive spatial-sequential WM (but not visual WM) is linked to mathematical abilities. The association between passive spatial-sequential WM and math that we found is consistent with previous studies showing an involvement of spatial-sequential WM in a wide range of mathematical abilities [14,57,58,69,70]. With regard to the passive visual WM component, some evidence suggested that this component is not involved in mathematical abilities [58,59]. This conclusion is, however, suggested by studies on children with mathematical difficulties. Indeed, children with mathematical difficulties, compared to TD children, fail in spatial WM tasks but do not fail in visual WM tasks [58]. Our results indicate that in TD children, passive visual WM does not seem to play a pivotal role in solving mathematical tasks. Notably, Fanari et al. [68] showed an association between visually active WM and mathematical abilities in TD children. Therefore, while visual active WM has been shown to be involved in mathematical skills ([68], but see [59]), our results indicate that passive visual WM does not seem involved in solving mathematical tasks in TD children. Interestingly, as stressed above, in our first correlation analysis, we found an association between passive visual WM and some mathematical skills. However, we failed to find any relationship between passive visual WM and mathematical skills after analyzing the correlations through the Network Analysis approach. Thus, it is possible that any association between passive visual WM and math reflects the influence of active high-control VSWM processes.

In addition, we found no associations between the passive spatial-simultaneous component and mathematical abilities. This result is consistent with some other findings that indicate there is no relationship between math and the passive spatial-simultaneous component [69]. At the same time, however, our data are inconsistent with data suggesting that the passive spatial-simultaneous component is involved in mathematical skills [52,56,57,58]. One possible explanation for the conflicting evidence concerns the task used to assess spatial-simultaneous WM in this current study. Indeed, spatial-simultaneous WM may be assessed through several tests, and all the studies supporting a link between spatial-simultaneous WM and math used tasks different from the one used in this present study. Notably, as stressed by Mammarella et al. [57], spatial simultaneous WM may interact and/or overlap with passive visual WM ‘since simultaneous presentation of locations could suggest a shape’ [57]. Thus, a minimal difference in the presentation of stimuli in different tasks that assess spatial-simultaneous WM could affect the validity of the test to evaluate the spatial-simultaneous WM component specifically. Thus, it is possible that compared to the tasks that measure the spatial-simultaneous component of WM used in previous studies, the task used in our study does not properly and specifically capture the spatial-simultaneous component. However, taking into account our results together with the data found by Allen et al. [69], we think that further studies need to explore the involvement of the spatial-simultaneous passive component of WM in mathematical skills.

It has been suggested that younger children rely more on VSWM when learning and applying new mathematical skills, whereas older children rely more on verbal WM (after skills have been learned). Accordingly, some studies indicate that the association between VSWM math abilities starts to decline from around 7–8 years [17,53,55,62]. Our results, however, suggest the possibility that low-control VSWM also keeps a role in solving math tasks in older children. Our findings regarding the association between VSWM and math in older children are consistent with many studies showing that (i) the association between VSWM and math abilities may result in stable across age [13,69,94,95] or become stronger as a function of age [14,94,95], and (ii) there may be a development related shift from verbal to visuo-spatial WM and related strategy used to solving some math tasks [45,50,70,71], as well as a development related shift from visuo-spatial to both visuo-spatial and verbal WM strategy used to solve math tasks [53,55,95]. Moreover, De Vita et al. [53] found that passive VSWM predicts early mathematical knowledge only among preschoolers, while active VSWM predicts math skills in both preschoolers and first-grade children. Thus, De Vita et al.’s study ([53]; see also [43]) suggests that the influence of low-control VSWM in solving math tasks begins to decline starting from the first grade of primary school, while high-control VSWM processes seem to play a pivotal role in both young and older children. Our data suggest the possibility that low-control VSWM keeps a role in solving math tasks also in older children (independently from the possible influence of high-control VSWM).

In summary, it is possible to highlight that our data indicate that both passive and active VSWM seem to be involved in math skills in (older) children, as well as that between the different passive components of VSWM only the spatial-sequential component seems to play a role in math abilities.

### 4.2. The Relationship between Different Mathematical Skills and Different VSWM Components and Processes

This study not only provides evidence concerning the relationship between passive and active VSWM and math but addressed also whether different mathematical skills have different relationships with the various VSWM processes and components in children. From the above discussion, it is clear that neither passive visual WM nor passive spatial-simultaneous WM seems involved in none of the specific math skills assessed in this study. At the same time, we found that each active VSWM and passive spatial-sequential WM are only associated with specific math skills. In particular, passive spatial-sequential WM was associated with written calculation and arithmetical facts, while active VSWM was associated with the ability to retrieve arithmetical facts and the ability to process the syntactic structure of numbers. Notably, we have not found an association between the number ordering task and any of the VSWM components. The number ordering task requires ordering Arabic numbers from the smallest to the biggest and vice versa. Considering the strong relationship between the semantic representation of numbers, spatial skills, and VSWM [11,14,65,67], this result was unexpected and needs to be explored in future studies.

Concerning the relationship between passive spatial-sequential WM and math abilities, it is important to stress that we found that the tasks used to assess passive spatial-sequential WM were, respectively, associated with two different math abilities (i.e., performance in Corsi blocks Task was associated with written calculation but not to arithmetical facts, while, on the contrary, performance to “sequential matrix task” was associated with arithmetical facts but not to written calculation). It is possible to observe that both Corsi blocks Task and written calculation involved a motor component, as well as a spatial representation, which may explain, at least in part, this association. In any case, our result indicates a possible relationship between passive spatial-sequential WM and both written calculation skills and arithmetical facts retrieval. With regard to written calculation skills, previous studies found that active VSWM was involved in subtraction with borrowing in TD children [64]. Moreover, several studies showed a relationship between passive spatial-sequential WM and written calculation in children with neurodevelopment disorders [57,58]. Our data provide new evidence that indicates that passive spatial-sequential WM supports written calculation in TD children. Caviola et al. [64] suggested that VSWM may support subtraction with borrowing by, for instance, retaining and completing the borrowing procedures. Mammarella et al. [57] found that errors (e.g., carrying error or column confusion) made by children with nonverbal learning difficulties in the written calculation were related to difficulties in spatial-sequential WM passive task performances. The written calculation involves precise spatial-sequential procedures, and, consequently, it needs spatial-sequential WM (i.e., procedures are sequential, and a precise order must be followed to arrive at the result).

Our data also indicate that passive spatial-sequential WM and active VSWM are associated with performance in arithmetical facts retrieval. This result is inconsistent with Dehaene’s model of mathematical cognition [11]. Indeed, this model suggests that arithmetical facts are stored and retrieved in a verbal form; therefore, retrieving the simple arithmetic facts is considered a language-based skill that does not require VSWM. Previous studies found a relationship between VSWM and arithmetical facts in children [16], but such findings have been interpreted depending on the format presentation (written format) of the task [16]. In our study, however, we found an association between VSWM and arithmetical facts, although arithmetic facts were orally presented. Therefore, our data cannot be explained by reference to the modality (i.e., visual) in which the arithmetic facts were presented. It is possible that the process of retrieving arithmetic facts from long-term memory may not be fully automated in some children; therefore, they have to use mental calculation procedures involving VSWM. In any case, the association that we found between VSWM and arithmetical facts is consistent with some recent studies indicating an implication of VSWM in the retrieval of arithmetical facts [7,16]. Thus, our study together with the results of other research encourages us to deepen the mechanisms that link arithmetic facts retrieval to the visuo-spatial processes.

Relatively few research addressed the implication of WM in number transcoding skills in children. However, some studies indicate that WM may play a pivotal role in number transcoding abilities in children [67,96,97]. Simmons et al. [67] found that transcoding ability is associated with passive VSWM but not with the CE component of WM. Notably, Simmons et al. assessed CE without distinguishing between the verbal and the visuospatial format. In our study, we found that active (but not passive) VSWM is associated with transcoding ability in children. This may suggest that VSWM plays a crucial role in the active manipulation of visual-spatial information regarding the digit chain and position necessary to number writing correctly.

Our study has several limitations. With regards to the associations between active VSWM and math, we cannot be sure that these associations do not reflect general executive resources as we have not used a test that evaluates verbal active WM. Moreover, we assessed only an active component of VSWM (i.e., active spatial-simultaneous WM), while C-WM suggests that each component may process information actively or passively (in other words, we did not assess active visual and active spatial-sequential components). Thus, other studies are required to acquire information regarding the relationship between math and active VSWM. Finally, further studies are needed in order to apply Network Analysis to assess whether the relationship between different VSWM components and processes and different math skills varies with age.

### 4.3. Conclusions

To conclude, our results indicate that both active VSWM and passive spatial-sequential WM are linked to (some but not all) math abilities, while other VSWM components (i.e., visual and spatial-simultaneous WM) appear to be not associated with any of the investigated math skills. Therefore, our result reveals that active VSWM and passive spatial-sequential (but not other VSWM components) are involved in mathematical tasks, particularly in specific math skills (i.e., written calculation, arithmetical facts, and in the elaboration of the syntactic structure of the number). Notably, it has been suggested that VSWM is mainly associated with tasks involving the processing of the analogical representation of quantities as well as in several mathematical domains that clearly rely on visual processing skills. Our study, however, highlights that VSWM appears to be implicated in several other mathematical skills, such as arithmetic facts and number syntax processing.

Data concerning the underlying processes involved in mathematical skills may be relevant for clinical and educational aims and may contribute to the development of effective interventions to improve mathematical abilities in typically developing children [98,99,100], children with mathematical learning deficits [101], and people with acquired mathematical disabilities resulting from brain damage [102,103]. Indeed, from the clinical and educational point of view, our results may suggest developing WM interventions that focus on training active VSWM and passive spatial-sequential components to improve some math skills.

## 5. Highlights

- Network Analysis is a flexible method for exploring the relationships between the different VSWM components/processes and several mathematical abilities.
- Both passive and active VSWM seem to be involved in math skills in (older) children.
- Between the different passive components of VSWM only the spatial-sequential component seems to play a role in math abilities.
- Diverse mathematical abilities have different relationships with the various VSWM processes/components.

## Figures and Tables

**Table 1:** The lower part (highlighted in dark grey) reports the simple correlations, measured with Pearson’s r. The upper part (highlighted in light grey) reports the network weights, which correspond to regularized partial correlations. The diagonal reports the mean z scores ± the standard deviations.

		Math				Working Memory				
		Facts	Ordering	Digits	Calculation	Balloons	Corsi	Matrices	Dots	A-VPT
Math	Facts	0.18 ± 1.01	0.13	0.12	0.25	0	0	0.02	0	0.15
	Ordering	0.36 ***	0.11 ± 0.99	0.07	0	0	0	0	0	0
	Digits	0.37 ***	0.30 ***	0.06 ± 0.89	0.04	0	0	0	0	0.09
	Calculation	0.47 ***	0.23 *	0.29 **	0.02 ± 0.84	0	0.05	0	0	0
Working Memory	Balloons	0.20 *	0.15	0.16	0.20 *	0.03 ± 1.06	0	0	0	0
	Corsi	0.15	0.05	0.20 *	0.25 **	−0.02	−0.11 ± 0.99	0	0	0
	Matrices	0.22 *	0.04	0.14	0.06	0.15	0.06	0.01 ± 1.07	0	0
	Dots	0.10	−0.09	0.01	−0.03	0.20 *	0.16	0.17	−0.11 ± 1.15	0
	A-VPT	0.37 ***	0.22 *	0.32 ***	0.25 ***	0.08	0.13	0.04	−0.03	−0.46 ± 1.98

* *p* < 0.05, ** *p* < 0.01, *** *p* < 0.001.

## Data Availability

The data presented in this study are available on request from the corresponding author. The data are not publicly available due to privacy and ethical reasons.
